# Fuzzy-set qualitative comparative analysis of factors affecting the use of e-cigarettes among college students in Guangdong province

**DOI:** 10.18332/tid/187836

**Published:** 2024-05-29

**Authors:** Xiaoyu Tan, Jianrong Mai, Lina Lin, Ling Zhou, Tingfen Huang

**Affiliations:** 1 Guangzhou Xinhua University, Guangzhou, China

**Keywords:** e-cigarette, college students, fuzzy set qualitative comparative analysis, self-efficacy

## Abstract

**INTRODUCTION:**

In recent years, e-cigarettes as an emerging tobacco product have been favored by college students. Our study aims to explore the factors affecting the use of e-cigarettes among college students and to put forward feasible suggestions for effectively controlling the use of e-cigarettes among college students.

**METHODS:**

The participating students were from three undergraduate and three specialized colleges in Guangdong Province, surveyed from January to March 2022. The Fuzzy-set Qualitative Comparative Analysis (fsQCA) method was used to analyze the influence mechanism and path of five antecedents: self-efficacy, social environment, cognition, sales environment, and negative outcome expectation, on the use of e-cigarettes. The fsQCA used in this study is a novel research methodology that combines the strengths of qualitative and quantitative analyses, through which we can determine which conditions are essential to the outcomes that lead to e-cigarette use among college students, and which combinations of conditions are more important than others.

**RESULTS:**

The interaction of self-efficacy, social environment, cognition, sales environment, and negative outcome expectation, affected college students’ use of e-cigarettes. Through the fsQCA method, it was found that self-efficacy alone constitutes a necessary condition for college students not to use e-cigarettes. There are four possible pathways for college students not to use e-cigarettes, with higher self-efficacy, correct cognition, and a healthy social environment influencing the most important combination of conditions for college students to use e-cigarettes.

**CONCLUSIONS:**

The use of e-cigarettes by students in Guangdong Province is the result of the synergistic effect of multiple factors. Tobacco control action suggestions focus on improving students' self-efficacy and paying attention to the combination of different factors to achieve more effective tobacco control.

## INTRODUCTION

In recent years, e-cigarettes have been favored by college students as an emerging product. In other countries, the prevalence of e-cigarettes is high among college students. Kelsh et al.^1^ surveyed 3754 college students in the Midwest of the US and found that more than half of the respondents (55.2%) had used e-cigarettes. Some scholars point out that 17.7–40.0% of college students in the US have tried or are using e-cigarettes. A study by Phetphum et al.^2^ involving 792 college students in northern Thailand found that 18.1% had used e-cigarettes in the

past 30 days despite a domestic ban on imported e-cigarettes. Although the college student’s e-cigarette use rate in China is lower than in other countries^3^, e-cigarette use and awareness among them continue to rise year after year, and since China has the largest production of e-cigarettes, the use of e-cigarettes among the Chinese college population is likely to grow rapidly in the future. In a survey of 3492 college students in Shanghai, Chen et al.^4^ found that the rate of e-cigarette use among college students was 7.65%. In a survey of 925 university students in Wuhan, Qiu et al.^5^ found that the awareness rate of e-cigarettes among university students was 80.6%. Peng et al.^6^ surveyed 2694 college students in Guiyang City and found that the rate of e-cigarette experimentation among college students was 4.90%. There have been studies showing that there are many reasons affecting college students’ use of e-cigarettes, mainly personal, product, environmental, and social factors. Peng et al.^6^ surveyed college students in Guizhou city and found that insufficient knowledge of e-cigarettes and positive attitudes increase the risk of students’ use of e-cigarettes, while Yang et al.^7^ surveyed college students in Wenzhou city and found that college students’ main reason for using e-cigarettes is that they like certain flavors, such as fruit flavors, etc. Xiong et al.^8^ found that e-cigarette advertising and marketing may influence college students’ e-cigarette attempts and use, and in addition, having a family member who smokes, peer effects, and college students’ use of e-cigarettes were significantly associated.

Policy management of e-cigarettes has become a common goal for all countries. The Measures for the Administration of Electronic Cigarettes (the Measures) issued by the China Tobacco Monopoly Administration (CTMA), which came into effect on 1 May 2022, establishes a framework for the full-scale regulation of e-cigarettes, including a comprehensive range of e-cigarettes, including the management of production and quality and safety of e-cigarettes, sales management, supervision and inspection, and other comprehensive content. It is noteworthy that the Measures have strengthened the protection of students: e-cigarette flavors are stipulated as ‘should not be promoted to minors, and should not make the characteristic flavor of the product other than that of tobacco’. Nowadays, e-cigarettes are only allowed to have tobacco flavors, which may reduce the possibility of students trying to use e-cigarettes due to the richness of flavors of e-cigarettes. In terms of advertising, the Measures explicitly prohibit the organization of various forms of exhibitions, forums, fairs, etc. to promote e-cigarette products, which means that it is basically difficult for the future e-cigarette market to increase the influence of a brand through marketing, which may, to some extent, reduce the rate of students’ awareness of e-cigarettes. In terms of sales methods, the sales of e-cigarettes will be more strictly audited, which can to a certain extent reduce the number of ways that students can buy e-cigarettes, such as micro-merchants in the circle of friends, online network sales, and so on.

Qualitative Comparative Analysis (QCA), first proposed by Ragin in 1987, is a Boolean algebra-based configuration analysis method that explores multiple complex causal problems through the relationship between research conditions and conclusions. This is in contrast to standard multiple regression analysis, which focuses on the single effect of variables on the dependent variable and the effect of key variables on the results. In contrast, Fuzzy-set QCA emphasizes that different paths can lead to the same complex combination and diversity of results and conditions and has been widely used in management^9^, sociology^10^ and other fields in recent years, but it is still rare in the field of public health.

This study uses the fsQCA method mainly based on the following three reasons: 1) Five antecedent variables were included in this study to explore how they interact in the future on e-cigarette use among college students. However, conventional statistical analyses relying only on a single factor or two-by-two factors cannot do this, so this study used the QCA method to analyze the combinations generated by these five antecedent variables and ultimately arrived at the core conditions, the borderline conditions, and the combinations of conditions by which they influence the results, and, through these pathways, to identify the facilitators buffering college students’ use of e-cigarettes; 2) The calibration of fsQCA can bridge the gap between qualitative and quantitative methods by converting the data to the range 0–1 instead of the non 0 or 1 case, improving the accuracy of the results; and 3) QCA identifies causal chains that may lead to ‘equivalence’ among college students for the same outcome in future e-cigarette use, i.e. it can identify different sets of antecedent conditions that have perfect equivalence with the explained outcome without conflict^11^. Therefore, compared with traditional statistical analysis methods, the fs-QCA method is more suitable for exploring factors influencing college students’ e-cigarette use, facilitating researchers to explore potential alternative relationships among conditions, providing a theoretical basis for subsequent interventions, and suggesting more realistic recommendations.

Specifically, we attempt to answer the following research questions: ‘What are the conditions that influence e-cigarette use among college students?’, ‘Which conditions play a more important role in it?’, and ‘What kind of matching and substitution relationships exist between them?’.

## METHODS

### Sampling and data collection

Students from three undergraduate and three specialized colleges in Guangdong Province were surveyed through the principle of stratified whole-group sampling, and the questionnaire was distributed to the college students in Guangdong Province through the Questionnaire Star platform. All participants signed an electronic informed consent prior to the survey and agreed to begin the survey. Electronic questionnaires were distributed from January to March 2022. A total of 1085 questionnaires were distributed, and 1047 valid questionnaires were returned, with a valid recovery rate of 96.5%. The final sample consisted of 1047 college students, including 342 (32.7%) males, 705 (67.3%) females, 503 (48.0%) junior college students, and 544 (52.0%) undergraduates. The mean age was 20.1 ± 1.5 years.

### Outcome measurements

We used the Electronic Cigarette Use Scale (ECUS) questionnaire developed by our group. The questionnaire consisted of 24 items in 6 dimensions: self-efficacy (8 items), social environment (6 items), cognition (4 items), sales environment (3 items), negative outcome expectation (2 items), and behavioral intention (1 item)^12^. According to the Likert 7-level scoring method, calculated from ‘strongly disagree (1 point)’ to ‘strongly agree (7 points)’, a higher total score represents a lower likelihood of e-cigarette use. The total Cronbach’s coefficient of the questionnaire in this study was 0.898, indicating good reliability.

### The fsQCA

The process of using fsQCA in this study is as follows: 1) Reviewing the previous literature and combining realistic factors to identify the variables investigated; 2) combine with theoretical studies to design questionnaires and collect data; 3) combining theoretical and practical situations, the variables were calibrated to form fuzzy sets with values ranging 0–1; 4) performing necessity analysis of a single condition and adequacy analysis of conditional configuration mainly include two indicators, consistency, and coverage, which are used to confirm the relationship of subsets, and for necessity analysis of a single condition, 0.9 is currently used as the criterion for consistency in most studies; adequacy analysis of conditional configuration, 0.8 is used as the recommended criterion for current research^13^ coverage, interpretive power used to judge subset relationships; and 5) Discuss the configuration of each condition and finally make corresponding suggestions.

### Variable calibration

The software is a fuzzy-set version of the qualitative comparison program developed by Charles Ragin, Kriss Drass and Sean Davey and is available as the fsQCA 3.0. Referring to the fsQCA Manual, Chinese and related classic literature^14^, the specific calibration procedure is as follows: five variables, including self-efficacy, social environment, cognition, sales environment, negative outcome expectation as, and behavioral intention, were converted into sets of 0–1 and calibrated according to thresholds: complete membership (1), intersection (0.5) and complete non-membership (0). The former dependent variables in this study included self-efficacy, social environment, cognition, sales environment, and negative outcome expectation, and the outcome variable was behavioral intention. According to existing studies, the criterion of setting the complete membership threshold to a value of 6, the crossing point threshold to 4, and the complete non-membership threshold to 3 is calibrated^14,15^. Outcome variables: behavioral intentions included ‘no use of e-cigarettes in the coming year’ and ‘use of e-cigarettes in the coming year,’ assigned values of 1 and 0, respectively, with a test level of 0.05. The raw and calibrated profiles of all variables are shown in Table 1.

**Table 1:** Original dimension and its corresponding calibration

*Dimension*	*Raw range*	*Raw mean*	*Calibrated mean*
Behavioral intention	0–1	-	-
Self-efficacy	1–7	6.27	0.90
Social environment	1–7	6.00	0.88
Cognition	1–7	5.75	0.84
Sales environment	1–7	3.27	0.37
Negative outcome expectation	1–7	2.15	0.17

## RESULTS

### Analysis of necessary conditions

Analysis of necessary conditions is mainly used to determine whether self-efficacy, social environment, cognition, sales environment, and negative outcome expectations can constitute the necessary conditions for outcome variables^9^. According to existing studies, consistency greater than 0.9 and coverage greater than 0.5 indicate that this condition is necessary for the results. Analysis of the necessary conditions for college students not to use e-cigarettes in the coming year showed that the consistency of the condition of self-efficacy was higher than the cutoff value of 0.9. The coverage was greater than 0.5, suggesting that the condition of self-efficacy is necessary to explain to college students not to use e-cigarettes in the coming year. The results are shown in Table 2.

**Table 2:** Results of single factor necessity analysis

*Condition variable*	*Consistency*	*Coverage*
Self-efficacy	0.915	0.973
Self-efficacy*	0.085	0.818
Social environment	0.886	0.967
Social environment*	0.114	0.892
Cognition	0.850	0.964
Cognition*	0.150	0.924
Sales environment	0.365	0.953
Sales environment*	0.635	0.961
Negative outcome expectation	0.164	0.906
Negative outcome expectation*	0.836	0.969

* Negative variable.

### Analysis of sufficient conditions

Based on the analysis of 1047 case samples, according to the actual situation, the consistency threshold and case threshold were set as 0.928 and 5, respectively. Table 3 shows the configuration analysis of four conditions formed for college students in Guangdong Province who did not use e-cigarettes.

**Table 3:** Conditional configuration of non-use of e-cigarettes among college students in Guangdong province

*Condition/combination*	*1*	*2*	*3*	*4*
Self-efficacy	⚫a	⚫	⚫	⚫
Social environment	⚫b		⚫	⚫
Cognition	c	●d	●	
Sales environment				●
Negative outcome expectation	⨂e	⨂		
Raw coverage	0.765	0.739	0.787	0.352
Unique coverage	0.461	0.281	0.045	0.002
Consistency	0.982	0.979	0.978	0.968
Overall solution coverage	0.871			
Overall solution consistency	0.977			

a Solid circles (⚫ and ●) indicate condition presence. b Large circles (⚫ and ⨂) serve as core conditions. c Blanks indicate that this condition has no significant effect on the results. d Small solid circles (●) serve as auxiliary conditions. e Large crossed circles (⨂) indicate condition absence.

It can be seen from Table 3 that four antecedent condition combinations may affect the use of e-cigarettes by college students, and the consistency of the four combinations is greater than 0.8, which meets the criteria for consistency, indicating that the four combinations are sufficient conditions to affect the use of e-cigarettes by college students. The overall consistency was greater than 0.8, indicating that these four paths could better explain the influencing factors of e-cigarette use among college students. The overall coverage rate was 0.871, indicating that these four pathways could explain about 87.1% of college students’ use of e-cigarettes. From the original coverage rate, the four combinations explained 76.5%, 73.9%, 78.7%, and 35.2% of the overall coverage, respectively, and the four combinations were simplified as follows:

*Combination 1*: college students who did not use e-cigarettes = higher self-efficacy AND healthy social environment AND higher negative outcome expectation.

*Combination 2*: college students who did not use e-cigarettes = higher self-efficacy AND correct cognition AND higher negative outcome expectation.

*Combination 3*: College students not using e-cigarettes = higher self-efficacy AND healthy social environment AND correct perceptions.

*Combination 4*: College students not using e-cigarettes = higher self-efficacy AND healthy social environment AND restricted e-cigarette sales.

## DISCUSSION

The fsQCA can combine real-world data and research framework to determine the pre-dependent variables and outcome variables, forming a combination path of multiple pre-dependent variables and outcome variables, which is particularly suitable for exploring the problem of causal complexity^16^. The results of this study showed that self-efficacy, social environment, cognition, sales environment, and negative outcome expectation were the main factors affecting the use of e-cigarettes by college students, and different configurations combined had different effects on the use of e-cigarettes.

Self-efficacy alone constitutes a necessary condition for college students not to use e-cigarettes, which is consistent with the conclusion that ‘self-efficacy has an inverse relationship with smoking risk’ proposed by Bidstrup et al.^17^. Previous studies have shown that students with high self-efficacy would take appropriate strategies to refuse smoking in the face of the influence of peer smoking. This suggests that increasing self-efficacy among college students may be a key task in effectively controlling e-cigarette use, and schools should regularly organize for college students to participate in special tobacco control courses to enhance self-efficacy and master the methods of refusing to use e-cigarettes.

Self-efficacy and social environment coexist, which is an important condition combination for college students to use e-cigarettes. Analysis of *Combinations* 1, 3, and 4 shows that when a combination of higher self-efficacy and healthy social environmental conditions appears, regardless of the advantages and disadvantages of the other three conditions, college students cannot use e-cigarettes, which is different from previous studies focusing on the impact of social environment on the use of e-cigarettes^18^. This suggests that in tobacco control action, it is necessary not only to pay attention to the influence of the social environment but also to the influence of students’ internal factors, that is, self-efficacy, to achieve better tobacco control results. Specifically, it is recommended that families, schools and governments should jointly carry out tobacco control efforts^19^. In the family, parents should pay attention to the physical and mental health development of their children, in addition to general positive psychological cues, it is also very important to standardize their smoking behavior^20^. Both parents should avoid using e-cigarettes and act as role models for their children to reduce the likelihood of their children using e-cigarettes^18^. In terms of schools, while strengthening the construction of smoke-free campus^21^, we should also give full play to the educational and motivational role of schools, carry out the health science popularization course of e-cigarettes^22^, implement the tobacco control action to teachers and students^23^, strictly supervise the tobacco environment around schools, build a healthy smoke-free environment for students, and improve students’ determination to refuse to use e-cigarettes^18^. The government should constantly improve the laws and regulations of e-cigarettes, standardized and even smoke-free management of e-cigarettes in specific places^24^. Studies have shown that countries that impose e-cigarette bans are less likely to have students using e-cigarettes than other countries^25^.

Cognition plays the role of alternative sales environment, negative outcome expectation, social environment, and even combined with other conditions under certain conditions. Alison et al.^26^ showed that college students were relatively unaware of hazardous substances in e-cigarettes. University studies is the period of establishing values, and college students’ awareness of e-cigarettes is directly related to whether they use e-cigarettes^27^. Therefore, colleges and universities are encouraged to conduct a series of lectures on e-cigarettes to equip students with reliable and correct information in anticipation of reducing the likelihood of e-cigarette use among college students^28^.

Sales environments tend to have a subtle impact on e-cigarette use. In general, restricting e-cigarette sellers near schools is beneficial in reducing students’ likelihood of using e-cigarettes^29^. In this study, we found that having high self-efficacy, being in a healthy social environment, and limiting e-cigarette sales were effective in reducing e-cigarette use among college students. Therefore, it is suggested that the government should continue to improve the relevant regulations to restrict the sale of e-cigarettes in the vicinity of schools^26^.

### Strengths and limitations

The theoretical contributions of this study include: 1) A new method was used, Fuzzy-set Qualitative Comparative Analysis (fsQCA), to reveal the intrinsic mechanisms that determine e-cigarette use among college students, providing empirical and theoretical evidence for the complexity of causality; 2) This study bridges the gap between previous related studies that explored the one-factor net effect of e-cigarette use among college students with traditional analytic methods and identifies multiple pathways that lead to the same outcome of e-cigarette use among college students. The proposed methodology contributes to the theoretical development of the field; and 3) Adaptation of the application of the fsQCA method in a new field and demonstration that it has great promise for application in the field of social health research and the health of specific groups as a whole.

The limitations of this study are: 1) The geographical limitation of the survey; this study is only based on college students in Guangdong Province, the results of the study may be geographically biased; in the future, we can further expand the scope of the sample of the study, to enhance the robustness and universality of the conclusions, and to arrive at a more comprehensive result; 2) This study is cross-sectional, and causal inferences cannot be made; 3) This survey used a self-administered questionnaire, which differed from the influencing factors selected in other studies, and it is hoped that a uniform and better questionnaire related to e-cigarettes will be established at a later stage; and 4) Limitations of QCA; QCA methods are somewhat subjective in calibration, may lack consideration of time and dynamic dimensions, etc.

## CONCLUSIONS

Self-efficacy, social environment, cognition, sales environment, and negative outcome expectations were the main factors affecting e-cigarette use among college students. Families and schools should improve college students’ perceptions, self-efficacy, and negative outcome expectations of e-cigarettes, and the government should strengthen legislation and supervision to provide a healthy social environment for college students and further standardize the sales environment of e-cigarettes to reduce the use of e-cigarettes by college students.

## Data Availability

The data supporting this research are available from the authors on reasonable request.
